# Space–time dynamics regression models to assess variations of composite index for anthropometric failure across the administrative zones in Ethiopia

**DOI:** 10.1186/s12889-022-13939-7

**Published:** 2022-08-15

**Authors:** Haile Mekonnen Fenta, Temesgen Zewotir, Essey Kebede Muluneh

**Affiliations:** 1 Department of Statistics, College of Science, Bahir Dar University, Bahir Dar, Ethiopia; 2 School of Mathematics, Statistics and Computer Science, University of KwaZulu-Natal, Durban, South Africa; 3 School of Public Health, College of Medicine and Health Sciences, Bahir Dar University, Bahir Dar, Ethiopia

**Keywords:** Adjusted relative risk, Spatiotemporal models, Dynamic models, Spatial autocorrelation, Queen contiguity, Neighborhood effect, Lag effect

## Abstract

**Background:**

A single anthropometric index such as stunting, wasting, or underweight does not show the holistic picture of under-five children's undernutrition status. To alleviate this problem, we adopted a multifaceted single index known as the composite index for anthropometric failure (CIAF). Using this undernutrition index, we investigated the disparities of Ethiopian under-five children's undernutrition status in space and time.

**Methods:**

Data for analysis were extracted from the Ethiopian Demographic and Health Surveys (EDHSs). The space–time dynamics models were formulated to explore the effects of different covariates on undernutrition among children under five in 72 administrative zones in Ethiopia.

**Results:**

The general nested spatial–temporal dynamic model with spatial and temporal lags autoregressive components was found to be the most adequate (AIC = -409.33, R^2^ = 96.01) model. According to the model results, the increase in the percentage of breastfeeding mothers in the zone decreases the CIAF rates of children in the zone. Similarly, the increase in the percentages of parental education, and mothers’ nutritional status in the zones decreases the CIAF rate in the zone. On the hand, increased percentages of households with unimproved water access, unimproved sanitation facilities, deprivation of women's autonomy, unemployment of women, and lower wealth index contributed to the increased CIAF rate in the zone.

**Conclusion:**

The CIAF risk factors are spatially and temporally correlated across 72 administrative zones in Ethiopia. There exist geographical differences in CIAF among the zones, which are influenced by spatial neighborhoods of the zone and temporal lags within the zone. Hence these findings emphasize the need to take the spatial neighborhood and historical/temporal contexts into account when planning CIAF prevention.

## Background

In the lowest administrative units like zones and districts, health indicators such as nutrition give information that is needed to improve residents' health and to address local health concerns in susceptible geographic areas [1]. Undernutrition is one of the leading causes of death in children [1–3] and it is a major threat to child health. In most of the previous studies, researchers were interested in the relationship between nutrition status and place of residence [4–8]. Moreover, their interest in spatial variability was mainly focused on the macro levels of geography such as countries, states, regions, and cities. But studies of undernutrition at the lower administrative level (zones in our context) have great practical benefits. Besides, those previous studies generally did not account for the potential dependencies of undernutrition on both time and space [4–10]. In other words, temporally close periods and geographically close areal units tend to have more similar responses than those far apart [11–14]. Most of the previous studies on the prevalence of undernutrition in Ethiopia have focused on a single conventional anthropometric index of stunting, underweight, or wasting [4, 8, 15–21], separately proposed by the World Health Organization (WHO) [22]. However, because these traditional indices of undernutrition may overlap, a child may exhibit evidence of having two or more of these traditional measures at the same time, they are insufficient for establishing the overall true burden of undernutrition among children under the age of five [4, 16–18, 23–29]. We, therefore, developed a composite index of anthropometric failure (CIAF) which might overcome these limitations through an aggregation of the common indices of undernutrition measures [15–18, 30]. Understanding the space–time patterns and the important covariates of undernutrition in terms of the composite index for anthropometric failure (CIAF) in the under-five children in Ethiopia is important for health resource allocation-related issues, which further helps to reduce the child health disparities and inequalities. Additionally, presenting the risk of those indicators at the lower administrative (zonal) level is helpful for a spatially targeted intervention. The space–time dynamic model was used to introduce the time, space, and space–time interaction, and unobserved influencing factors, and thus provide better estimates of the relationships between undernutrition and the risk factors of known covariates [13, 14, 31–33]. As far as our knowledge is concerned, there is no study exploring the spatiotemporal patterns of CIAF risk in Ethiopian administrative zones. Hence, we propose a space–time dynamic model for undernutrition to estimate the space–time effects of covariates. Moreover, this study aimed to examine the patterns and identify the influencing covariates of CIAF in the Ethiopian administrative zones over the study period (2000–2016), using the EDHS data with the application of space–time dynamic models.

## Methods and statistical analysis

**Fig. 1:**
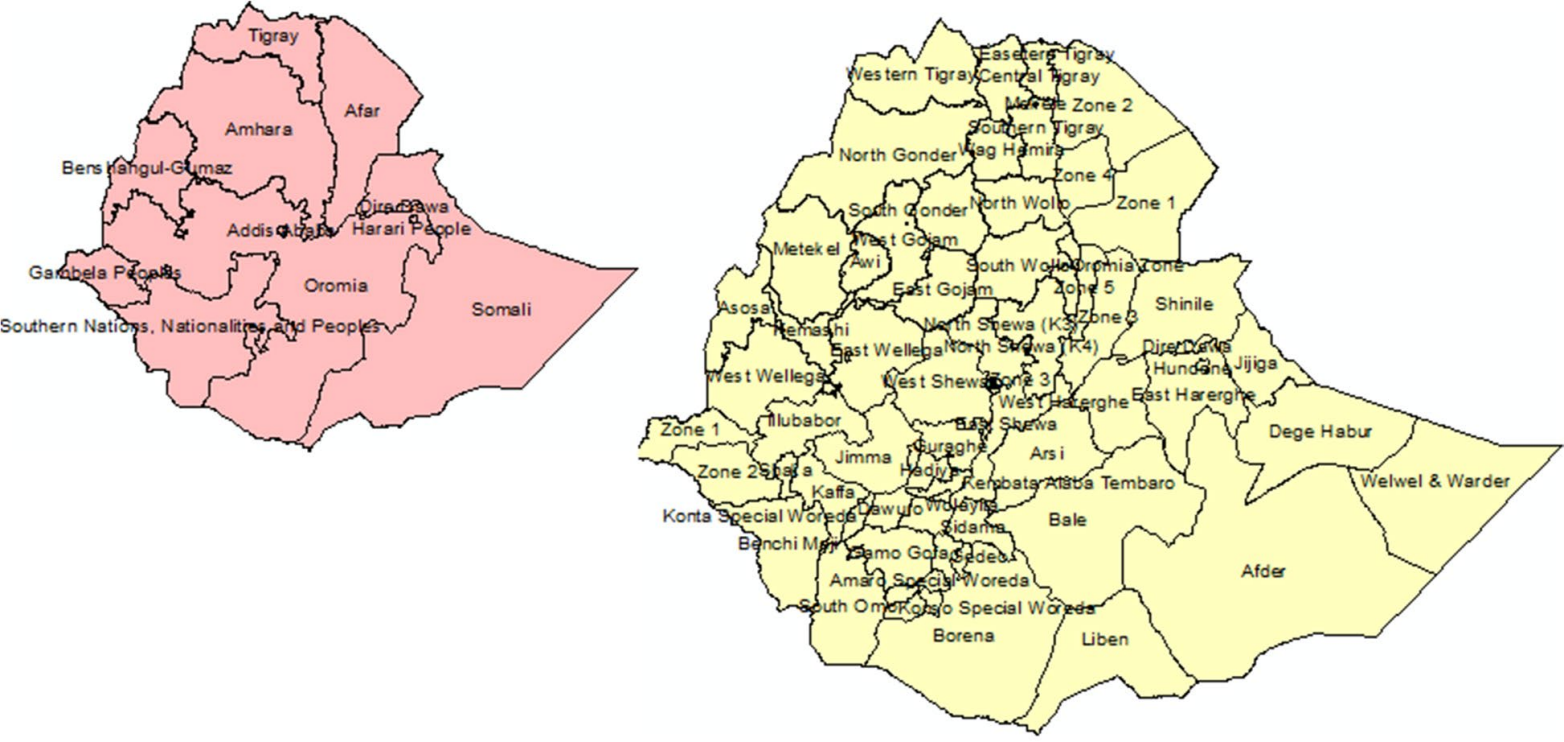
Locations of the 72 administrative divisions (zones) of Ethiopia: **a** Regions; **b** administrative zones of the study area (Source: Authors)

Data for the analysis was drawn from 72 administrative zones in Ethiopia. Ethiopia is located in East Africa (Fig. 1), with a total land area of 1.1 million km^2^. The country has 11 national regions and 72 administrative divisions (zones).

The country has undertaken several economic development programs across regions and zones for eradicating undernutrition, poverty, hunger, illiteracy, and infant and maternal mortality, among others. Despite all these efforts by the concerned bodies, there are economic or poverty disparities and inequalities between the different administrative zones of Ethiopia [34]. We used the secondary Ethiopian Demographic and Health Survey (EDHS). There are several EDHS datasets and for this study, we used birth history records. A total of 30,791 children consisting of 8,765 from 2016, 9,611 from 2011, 3,850 from 2005, and 8,565 from the 2000 EDHS respectively were plausible for analysis.

### Variables of the study

In this study, the zones are the spatial unit of analysis [13]. The outcome variable in this study was the proportion of CIAF for the zones [34]. Most of the previous studies on the prevalence of undernutrition in Ethiopia have focused on a single conventional anthropometric index of stunting, underweight, or wasting [4–8, 12, 19–21], separately proposed by the World Health Organization (WHO) [10]. However, these conventional indices of undernutrition may overlap so that the same child could show signs of having two or more of the indicators simultaneously; insufficient for determining the overall real burden of undernutrition situations among under-five children [5–7, 11–18]. The CIAF is computed by grouping those children whose height and weight were above the age-specific norm (above -2 z-scores) and those children whose height and weight for their age are below the norm and those who are experiencing one or more forms of anthropometric failure as express as B-wasting only, C-wasting and underweight, D- wasting, stunting and underweight, E- stunting and underweight, F-stunting only and Y- underweight only. The CIAF is then calculated by aggregating these six (B-Y) categories [16, 18, 27–29]. The choice of the covariates is guided by existing literature to study the determinants of child undernutrition in developing countries [4, 8, 10, 35]. In this paper, these explanatory variables considered in this study are also measured at the zone level. The zone-specific information on children, and households, such as the availability of improved drinking water, the percentage of literate mothers, the proportion of working mothers, and the percentage of households having access to drainage and sanitation facilities in the zones, was modeled with CIAF. The variables have been classified into the following categories: child, maternal, household, and geographic variables (Table 1).

**Table 1:** The description of the covariates included in the model

Childhood undernutrition using CIAF (outcome variable)	$$\mathrm y\mathrm i=\left\{\begin{array}{c}1:\mathrm{if}\;\mathrm{a}\; \mathrm{child}\; \mathrm{i}\; \mathrm{had}\;\mathrm{at}\; \mathrm{least}\;\mathrm{one}\; \mathrm{form}\; \mathrm{of}\; \mathrm{undernourished}\; \left(\mathrm {CIAF}\right) \\ 0: \mathrm{if}\;\mathrm{child}\; \mathrm{i}\; \mathrm{is}\; \mathrm{nourished}\; \end{array}\right.$$
**Child level covariates**	**Descriptions**
% of children with vitamin A	the proportion of children with vitamin A
% of children with breastfeeding	the proportion of children with breastfeeding
% of a child with comorbidity status	the proportion of children with comorbidity
% of children with a Dietary diversity score	the proportion of children with at least minimum dietary diversity score
**Maternal/household-level covariates**	**Description**
% of women with illiteracy	the proportion of women with an illiteracy rate
% of a father with illiteracy	the proportion of fathers with an illiteracy rate
% of women with high autonomy	the proportion of women with low autonomy
% of access sanitation facilities	the proportion of households with improved sanitation
% access to safe water	the proportion of households with improved water
% of women’s bmi < 18.5 kg/m2	the proportion of women with underweight BMI
% of women with media exposure	the proportion of women with media exposure
% of the working status of the mother	the proportion of women with working status
% of wealth Quantile (WQ)	the proportion of households with a high poverty rate
**Geospatial covariates**	**Description**
Average Precipitation (precp)	The average precipitation measured within the 10 km (rural) or 2 km (urban)
Average Aridity index	The ratio of annual precipitation to annual potential evapotranspiration (10 km × 10 km)
Average maximum temperature (MaxT)	The average annual maximum temperature within the 10 km (rural) or the 2 km (urban)
Average minimum temperature (MinT)	The average annual minimum temperature within the 10 km (rural) or the 2 km (urban)
Average potential evaporation (pet)	The average annual pet within the 10 km (rural) or the 2 km (urban)
Average urban–rural settlement (UR)	This is the urban–rural population classification of the area within the 10 km (rural) or the 2 km (urban)
Average Enhanced Vegetation Index (EVI)	The average vegetation index value within the 10 km (rural) or the 2 km (urban)
Average Wet days (WetD)	The average number of days receiving rainfall within the 10 km (rural) or 2 km (urban)

Different studies [1–5] showed that children from “arid” geographic areas were associated with undernutrition. In Ethiopia, we wanted to see the impacts of the change of geographical covariates on undernutrition [3–5]. This is because of frequent and severe shortfalls in precipitation, and continuous rises in temperature, which may result in food insecurity, droughts, and undernutrition. Furthermore, more than three-quarters of Ethiopians depend on subsistence and rain-fed farming, livestock production that is historically linked to low crop production, and less diversified and commercial foods. Therefore we have extracted the geospatial covariates from the GPS dataset of the demographic and health survey data and this is joined with the DHS row dataset. Finally, we successfully modeled the CIAF at the zonal level by using both the EDHS and geospatial covariates.

### Statistical methodology

The classical linear models estimated by ordinary least squares methods cannot take into account the fact that data collected based upon spatial and time specifications is not independent of its spatial location across different periods. If the spatial and temporal effects are neglected in the model, the estimated values will be biased [4–11, 36–40]. Observations available across space (N spaces) and time (T time points), a range of different model specifications need to be considered to allow different combinations of the two cases.

Let ${{\varvec{y}}}_{t}$ denote an NT $\times$ 1 column vector of observations on the dependent variable with spatial units (i = 1,2,..., N) and temporal units (t = 1,2,..., T), **X** be an NT $\times$ k matrix of observations on the covariates, and the spatial weight matrix **W,** which is constant over time, is the N $\times$ N positive matrix describing the spatial arrangement of the n units whose diagonal elements are set to be zero. Each entry ${w}_{ij}\in {\varvec{W}}$ represents the spatial weight matrix associated with units i and j [38–42]. The elements of ${w}_{ij}$ is (i, j), which is the neighborhood matrix of the row standardized matrix with a dimension of 72 $\times$ 72. Hence, the non-zero elements of the matrix indicate whether the two locations are neighbours. This weighted matrix is commonly expressed as:

$$w_{ij}=\left\{\begin{array}{cc}1&\mathrm{if}\;\mathrm{areas}\;\mathrm i\;\mathrm{and}\;\mathrm j\;\mathrm{are}\;\mathrm{neighours}\\0&\mathrm{otherwise}\end{array}\right..$$

The existence of spatial autocorrelation in the dataset is checked by using Moran’s I. The Moran’s I is used to associate weight (w_ij_) to each of the pairs [261–265], which quantifies the spatial pattern. The test is given as follows,

$$I=\frac{n}{{S}_{0}}\frac{\sum_{ij}({w}_{ij}({x}_{i}-\mu )({x}_{j}-\mu ))}{\sum_{i}{({x}_{i}-\mu )}^{2}}$$

where n is the number of investigated points, x_i_,x_j_ the observed value of two points of interest, $\mu$ the expected value of x, and w_ij_ the elements of the spatial weight matrix. In Moran’s I ranges [-1, 1] the value of 1 signifies that clusters with high values of the variable of interest are close to clusters with similar high values, while -1 indicates that high values are near to low values.

**Fig. 2:**
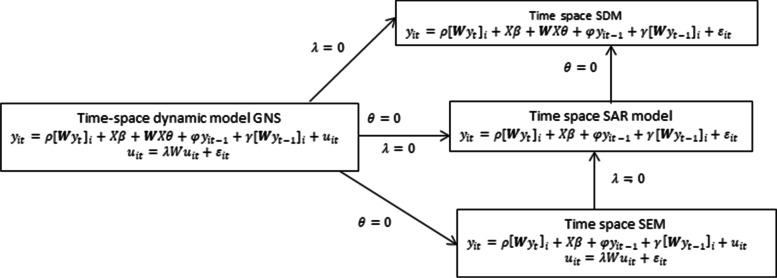
The space–time dynamic models. GNS: General Nesting Spatial model; SDM: Spatial Durbin Model; SAR: Spatial Autoregressive model; SEM: Spatial Error Model.

In this paper, the four basic spatial time dynamics models (spatial Durbin model, spatial autoregressive model, spatial error model, and general nested model with space–time), were adopted [14, 42, 43]. Let the **WX** be the interaction effects among the covariates with the spatial components, and the **Wu** the interaction effects among the error terms of different observations, ${[{\varvec{W}}{{\varvec{y}}}_{t}]}_{i}$ is the i^th^ element of the spatial lag vector in the same period. The ${[{\varvec{W}}{{\varvec{y}}}_{t-1}]}_{i}$ is the i^th^ element of the spatial lag vector of observations on the response variable in the previous time. When the response variable is related to the same locations as well as the neighboring locations in another period, the model is called a space–time recursive model. The ${y}_{it-1}$ is the observations on the dependent variable in the previous period. Moreover, let $\rho$ be the spatial dependence parameter, $\theta$ the spatio-temporal diffusion parameter, and $\phi$ the autoregressive time dependence parameter [4–11, 36–40] (Fig. 2).

When the response variable is related to the same locations as well as the neighboring locations in another period, the model is called the space–time recursive model. The ${y}_{it-1}$ is the observations on the dependent variable in the previous period. The standard assumptions that ${\varepsilon }_{ij}\sim N(0, {\sigma }^{2})$ and $E\left({\varepsilon }_{it}{\varepsilon }_{js}\right)=0$ for $i\ne j$ or $t\ne s$ apply in any case [12, 14, 36, 42, 43].

## Results

Table 2 summarizes the different measures of undernutrition status in Ethiopian children aged 0–59 months in the years 2000, 2005, 2011, and 2016. In the 2000 EDHS, 61.38% of children had one or more kinds of undernutrition (CIAF). According to established measures of undernutrition, 38.3%of children in the 2016 EDHS were stunted, 10% were wasting, and 23.3% were underweight. Moreover, around 46.49% of the children had at least one form of traditional undernutrition measures (Groups B-Y). For 2016 EDHS, the highest prevalence of undernutrition was found to be in Group F (19.47%) followed by Group E(15.76%), while Group Y was observed to be the lowest with respect to undernutrition (1.16%).

**Table 2:** Classification of undernourished under-five children and their percentages over time in Ethiopia

Group	Description of the group	Definition	Wasting	Stunting	Underweight	2000	2005	2011	2016
A	No anthropometric failure	Normal WAZ, HAZ, and WHZ	No	No	No	38.62	43.42	48.42	53.51
B	Wasting only	WHZ < -2SD but normal WAZ and HAZ	Yes	No	No	1.14	2.36	2.66	3.69
C	Wasting and underweight	WHZ and WAZ < -2SD but normal HAZ	Yes	No	yes	4.08	4.07	3.22	3.34
D	Wasting, underweight, and stunting	WHZ, WAZ, and HAZ < -2SD	Yes	Yes	Yes	5.50	4.10	3.97	3.08
E	Stunting and underweight	HAZ and WAZ < -2SD but normal WHZ	No	Yes	Yes	32.65	26.82	20.24	15.76
F	Stunting only	HAZ < -2SD but normal WAZ and HWZ	No	Yes	No	13.07	15.58	20.12	19.47
Y	Underweight only	WAZ < -2SD but normal HAZ and WHZ	No	No	Yes	4.87	3.50	1.37	1.16
Stunting	D + E + F	Height-for-age (HAZ < -2SD)				51.22	46.50	44.30	38.30
Wasting	B + C + D	weight-for-height (WHZ < -2SD)				10.70	10.50	9.90	10.10
Underweight	C + D + E + Y	Weight-for-age (WAZ < -2SD)				47.10	38.50	28.80	23.30
CIAF	B + C + D + E + F + Y (1-A)	Composite Index of Anthropometric Failure (CAIF)				61.38	56.58	51.58	46.49

From time to time, except for stunting only, the prevalence of all anthropometric measures was declined. Moreover, the CIAF was higher than the other measures, which indicated the real burden of the child's under-nutrition status in the country. Moreover, compared with 2000 EDHS, children in the years 2005, 2011, and 2016 were associated with lower the values of CIAF by 12.4%, 23%, and 29.7% respectively (Table 2).

**Fig. 3:**
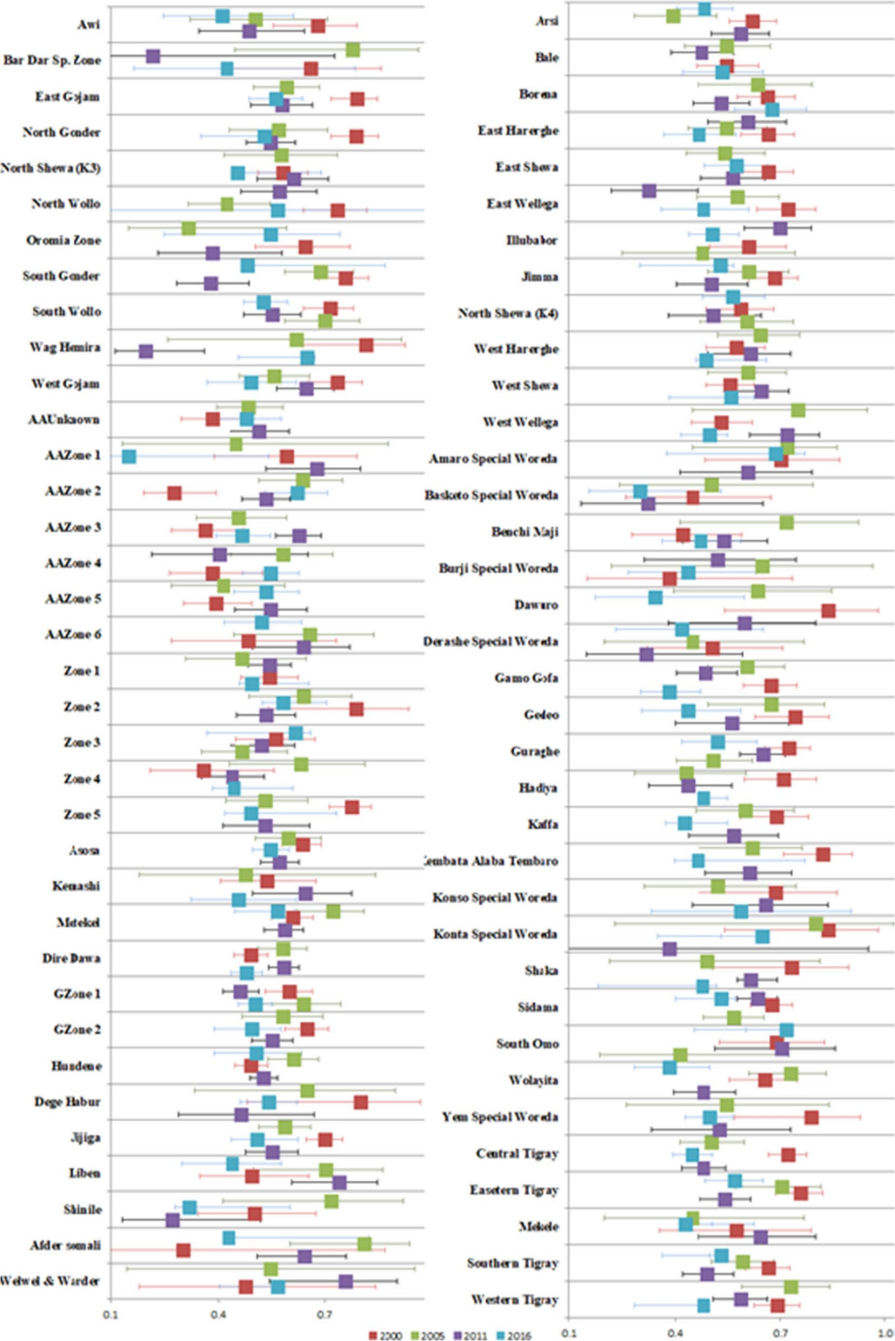
Observed prevalence of CIAF at zone-level among children under five years old in Ethiopia by survey years

The data for 72 Ethiopian administrative zones were considered for four consecutive EDHS (2000, 005, 2011, and 2016) for each wave. The data of CIAF and the risk factors from the 72 administrative zones of the country were aggregated to provide zone-level summaries over time. The observed value of CIAF varied with time, and Fig. 3 shows the temporal variations in the CIAF from 2000–2016 (the higher value occurs in 2000).

The observed prevalence with a 95% confidence interval which is adjusted by survey weight is shown in Fig. 3 for the 72 Ethiopian administrative zones. The observed value of CIAF generally decreased in 2016 which also showed less heterogeneity between zones, while it varied among zones in 2000, 2005, and 2011, with several zones showing high prevalence (> 50%). The zones with very high observed proportion (> 50%) included Dawuro, Wag Hemira, and Dege Habur in 2000; Amaro Special Woreda, West Wellega, and Konta special Woreda in 2005; and Yem Special Woreda, Liben, and West Wellega in 2011 (Fig. 3).

**Fig. 4:**
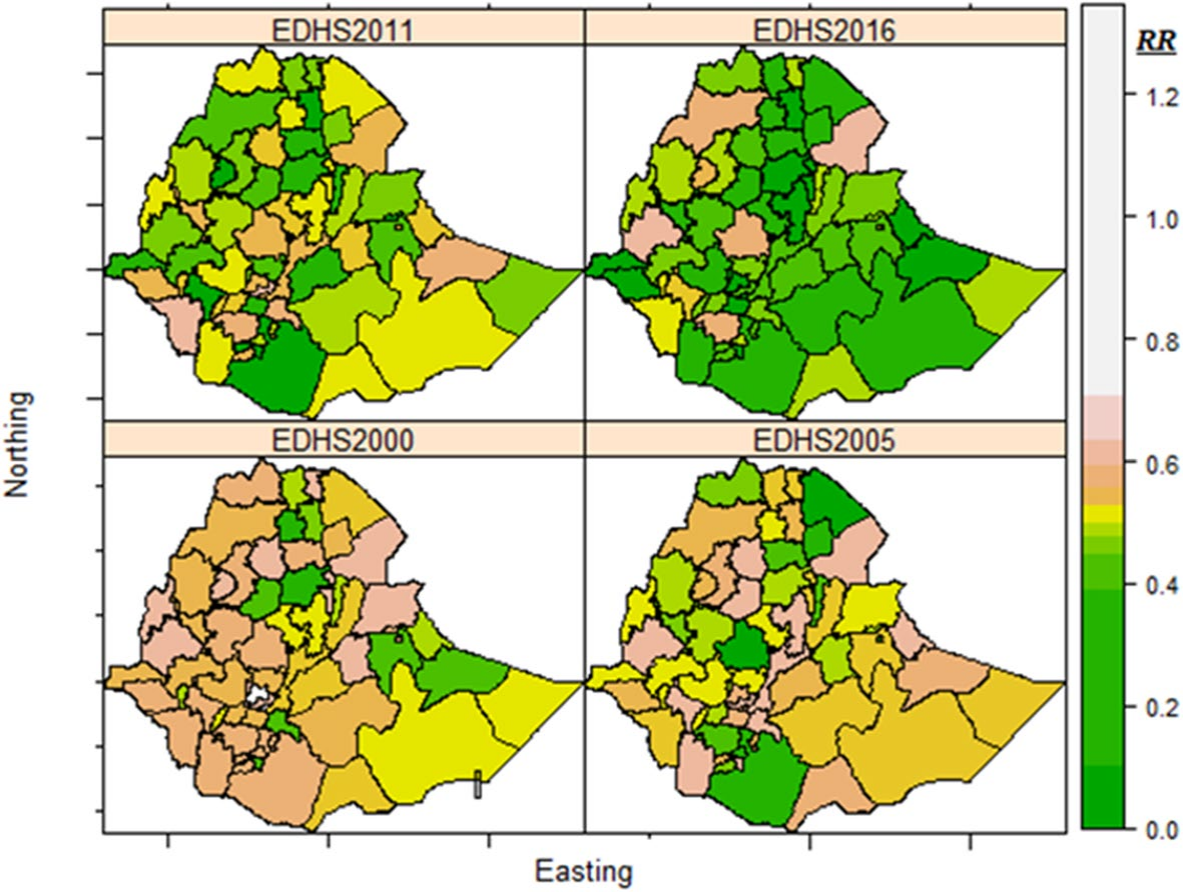
The estimated relative CIAF risk in Ethiopian administrative zones from 2000 to 2016

The spatial and temporal patterns of CIAF have been pictorially presented in Fig. 4. Overall, there is a great variation in the time trends across zones, suggesting inequalities and disparities in the rates of change in CIAF within the country over time. The diagram shows that all zones had the steepest improvement in CIAF over the study period. Moreover, the map suggests the existence of both spatial and temporal dependence structures in the CIAF relative risks. As can be seen from Fig. 4, by 2000 the CIAF was high in almost all zones of the country. On the other hand, the results of 2016 showed low relative risks of CIAF with less heterogeneity between zones.

The units of analysis were the zones, hence the results are entirely dependent on the aggregated zonal level data. The average proportion of the CIAF rate of the 72 Ethiopian zonal communities was 52%. The average percentage of mothers’ illiteracy in 72 Ethiopian zones was 73%, the average percentage of the community in the zones with no improved water and no improved toilets was 38.44% and 59.80% respectively. The coefficients of variation for urban–rural settlements, evi, dietary diversity score, wealth index, and working mothers, were high; showing wide variations among zones in Ethiopia. Significant autocorrelation was observed for both the CIAF and most of the independent covariates, indicating that CIAF and the covariates were highly spatially correlated (Table 3).

**Table 3:** Descriptive statistics of the selected indicators covariates and the Moran’s I test statistic

characteristics /variables	minimum	maximum	1^st^ quartile	Median	mean	3^rd^ quartile	SD (CV%)	Moran’s I values
% under-five nutrition (CIAF)	33.87	67.74	47.86	52.89	51.99	55.52	6.2 (11.93)	0.034 ***
% children were vitamin A	49.69	72.13	44.24	50.31	49.69	53.94	8.1 (16.30)	-0.014
% children with breastfeeding	51.06	85.29	66.12	71.00	70.45	75.05	6.32 (8.97)	0.006**
% children with comorbidity	16.04	52.94	28.90	32.62	32.67	35.53	6.4 (19.59)	0.264*
% dietary diversity score (dds)	10.37	44.64	20.57	24.46	24.90	28.91	10.4 (41.77)	-0.014
% women with illiteracy	51.799	93.55	66.78	73.29	72.36	78.50	9.33 (12.89)	0.006***
% father with literacy	29.03	84.37	49.13	54.44	54.72	61.64	10.63 (19.43)	0.190***
% of women with autonomy	27.12	64.77	41.56	45.14	46.20	51.61	7.98 (17.27)	-0.014***
% access sanitation facilities	11.76	70.50	29.57	37.48	38.44	48.07	12.11 (31.50)	0.006***
% access to safe water	32.35	82.49	53.55	59.99	59.80	66.10	9.65 (16.14)	0.070**
% of women’s bmi < 18.5 kg/m2	4.84	50.00	20.72	24.42	24.23	27.04	6.53 (26.95)	-0.014
% media	14.44	61.36	30.93	37.28	36.40	42.01	9.78 (26.87)	0.006***
% working women	9.09	61.29	29.06	35.74	35.43	42.24	10.25 (28.93)	0.155
%wealth	0.00	82.26	32.33	40.44	41.21	51.24	16.76 (40.67)	-0.014*
mean of precipitation	58.94	116.65	77.59	90.45	88.28	98.27	13.90 (15.75)	0.006*
mean aridity	13.75	33.01	19.33	23.52	23.41	25.79	4.79 (20.46)	0.117*
mean evi	-2322	2390.78	882.68	1409.63	1259.05	1728.74	798.7 (63.44)	-0.014
mean eleviation	1.81	7.70	2.87	3.54	3.73	4.20	1.14 (30.56)	0.006***
mean maximum temperature	24.70	31.62	27.12	28.10	28.25	29.25	1.44 (5.10)	-0.093**
mean minimum temperature	9.85	17.88	12.54	13.46	13.78	15.04	1.67 (12.12)	-0.014**
mean pet	3.47	4.66	3.83	4.05	4.05	4.28	0.27 (6.67)	0.005
mean ur	0.001	0.27	0.013	0.023	0.037	0.045	0.027 (72.97)	0.505**
mean wetd	5.60	8.64	6.62	7.28	7.24	7.77	0.76 (10.50)	-0.014 ***

*SD* Standard deviation, *CV* Coefficient of variation, *, ** and *** = Moran’s I are significant at 10, 5, and 1% level

The estimated parameters of the models were given in Table 4. Different space–time dynamic models for the period between 2000–2016 were fitted, by considering both the time, space, and their interactions as well to determine the relationships between the CIAF and different levels of covariates. The result reports the estimates of the parameters with a 95% confidence interval.

**Table 4:** The parameter estimation with 95% CI for spatial and temporal models to explain CIAF

characteristics	SDM	SAR	SEM	GNS
% children with breast feeding	-0.60 (-1.08, -0.11)	-0.22 (-0.50,0.06)	-0.25 (-0.52, 0.02)	-0.45 (-0.66, -0.25)***
% children without comorbidity	-0.62 (-1.13, -0.11)	-0.11 (-0.35,0.13)	-0.14 (-0.37, 0.09)	-0.53 (-0.74, -0.32)**
%dds minimum and above	-0.41 (-1.17, 0.35)	-0.43 (-0.74,-0.12)*	-0.50 (-0.81, -0.19)**	-0.13 (-0.46, -0.20)**
% children was not vitamin A	-0.31 (-0.76, 0.13)	0.05 (-0.19,0.30)	0.05 (-0.19, 0.28)	-0.63 (-0.84, -0.41)**
% women with illiteracy	0.54 (-0.06, 1.15)	0.28 (0.03,0.52)*	0.30 (0.06, 0.54)*	0.43 (0.17, 0.68)***
% father with literacy	-0.09 (-0.92, 0.74)	0.02 (-0.21,0.25)	0.00 (-0.23, 0.23)	0.04 (-0.30, 0.39)
%authonomy (low autonomy)	0.11 (-0.26, 0.49)	-0.05 (-0.25,0.14)	-0.05 (-0.24, 0.14)	0.29 (0.12, 0.45)*
% not access sanitation facilities	0.23 (-0.12, 0.57)	-0.11 (-0.30,0.07)	-0.11(-0.30, 0.07)	0.32 (0.18, 0.47)**
% no access to safe water	0.11 (-0.13, 0.35)	0.09 (-0.04,0.21)	0.11 (-0.02, 0.24)	0.20 (0.09, 0.30)*
% of women’s bmi > 18.5 kg/m2	-0.12 (-0.90, 0.65)	-0.29 (-0.64,0.05)	-0.30 (-0.63, 0.04)	-0.52 (-0.87, -0.17)*
media	0.11 (-0.52, 0.73)	-0.28 (-0.50,-0.05)**	-0.29 (-0.51, -0.07)*	-0.14 (-0.42, 0.13)
% working women	0.24 (-0.31, 0.78)	0.17 (-0.04,0.37)	0.20 (0.00, 0.40)	0.57 (0.31, 0.82)**
wealth (rich and richest)	-0.27 (-0.47, -0.08)*	-0.19 (-0.30,-0.07)	-0.19 (-0.30, -0.08)**	-0.36 (-0.45, -0.27)***
precipitation	0.03 (0.00, 0.06)	0.01 (0.00,0.02)	0.01 (0.00, 0.02)	0.04 (0.03, 0.05)**
aridity	-0.06 (-0.16, 0.04)	-0.04 (-0.08,0.01)	-0.03 (-0.07, 0.01)	-0.11(-0.15, -0.07)**
evi	0.00 (0.00, 0.00)	0.00 (0.00,0.00)	0.00 (0.00, 0.00)	0.00 (0.00, 0.00)
eleviation	0.02 (-0.03, 0.06)	-0.01 (-0.02,0.01)	0.00 (-0.02, 0.02)	0.00 (-0.02, 0.02)
max	-0.02 (-0.16, 0.12)	-0.03 (-0.09,0.02)	-0.03 (-0.08, 0.03)	-0.10 (-0.16, -0.04)*
mint	0.03 (-0.08, 0.13)	0.04 (-0.01,0.08)	0.03 (-0.02, 0.07)	0.08 (0.04, 0.13)*
pet	-0.07 (-0.41, 0.28)	-0.18 (-0.39,0.03)	-0.19 (-0.39, 0.01)	0.01 (-0.14, 0.15)*
ur	-0.33 (-1.29, 0.63)	-0.27 (-0.64,0.11)	-0.32 (-0.69, 0.05)	0.49 (0.00, 0.98)
wetd	-0.14 (-0.28, 0.01)	-0.03 (-0.09,0.03)	-0.03 (-0.09, 0.03)	-0.16 (-0.22, -0.11)
intercept	1.05 (-1.64, 3.74)	2.04 (0.50,3.59)*	2.12 (0.62, 3.62)**	1.13 (0.02, 2.23)*
$\rho$	-0.23 (-0.73, 0.27)	0.04 (-0.02,0.09)	0.04 (-0.02, 0.09)	-0.01 (-0.23, 0.21)
$\gamma$	-0.24 (-0.46, -0.03)	-0.02 (-0.07,0.02)	-0.03 (-0.07, 0.02)	-0.20 (-0.29, -0.12)**
$\lambda$			-0.19 (-0.41, 0.02)	-1.65 (-2.24, -1.07)**
$\phi$	0.05 (-0.29, 0.39)	0.10 (-0.05,0.26)	0.10 (-0.05, 0.25)	0.21 (0.06, 0.36)*
AIC	-270.35	-175.20	-179.08	**-409.33**
R2 (adjusted)	76.43	66.09	67.89	**96.01**
N	72	72	72	72

^*, ** and ***^ Significant at 0.05, 0.01 and 0.001 level of significance

The results show different spatio-temporal dynamic models of neighborhood contexts and CIAF of under-five children in Ethiopia. According to the AIC (AIC = -409.33), the lowest statistic and the higher R^2^ adjusted values indicated is the more appropriate statistical model, which suggests that using the general nesting space–time dynamic model is superior to other models in characterizing the undernutrition (CIAF) status of the under-five children in the 72 administrative zones in Ethiopia, as shown by the smallest values. The significant and positive temporal dependence ($\gamma$) indicated that past undernutrition tends to produce future sustained undernutrition status. Both the previous year and same place effects ($\varphi$) and the corresponding spatial parameter ($\lambda$) are statistically significant for the undernutrition status of the under-five children. All the covariates were standardized before the model was fitted so that the interpretation of the odds (relative risks) is expressed based on a one-standard deviation (SD) increase in the standardized covariates. The chosen dynamic model revealed significant child, household, and geographical covariates.

The coefficients from the spatial-time dynamic model indicated that zones with a higher percentage of breastfeeding are negatively associated with lower CIAF (Spatial GNS lag: B = -0.45, *p* < 0.001). The zones with higher percentages of a child without comorbidity are negatively associated with higher CIAF status (Spatial GNS lag: B = -0.53, *p* < 0.001). Moreover, zones with a higher illiteracy rate of mothers are also positively associated with higher CIAF percentages (Spatial GNS lag: B = 0.43, *p* < 0.001). Moreover, the regression coefficients of breastfeeding rate, minimum and above dietary diversity score rate, presence of comorbidity rate, and wealth index rate, were negative. This indicated that the variables were associated with a decrease in the incidence of undernutrition in terms of CIAF. However, the regression coefficients of the rate of women’s illiteracy, women’s low autonomy, and having no access to improved water and sanitation were positive, indicating that these factors were associated with an increase in the risk of under-five children undernutrition in the Ethiopian administrative zones (Table 4).

## Discussion

Childhood undernutrition is a major public health concern in Ethiopia [4, 10, 34, 44, 45]. Undernutrition (CIAF) in Ethiopia decreased from 61.38% to 46.49% for under-five children respectively, between 2000 and 2016. Various space–time dynamics models have been used for reducing spatial autocorrelation in model residuals. Our findings identified variations in the undernutrition of children under five among the 72 administrative zones in Ethiopia over the periods from 2000 to 2016. Four spatial–temporal dynamic models were used to evaluate the relationships between the CIAF and its covariates. In the modeling process, we sought to select the best model by considering the evaluation criteria of the models such as R^2^ and AIC. The result showed that when the spatial weight and spatial lag weight matrix were added in the GNS model, the adjusted R^2^ was maximized and AIC was minimized. This result is reasonable because neighboring zones may have effects on each other through sharing similar dietary and living habits, and environmental conditions, and the like. Both the observed and model-based estimated relative risks showed a decrease of CIAF risk from 2000 to 2016 in most of the Ethiopian administrative zones which is similar to what has been reported in different countries [46–48, 3–5] . Even though overall decreasing temporal trend of CIAF in Ethiopia is encouraging, the local trends have shown apparent heterogeneity. This is due to the fact that different administrative zones have their own cultural practices towards nutrition and even the local administrators have different commitments to the implementation of rules and regulation to minimize the undernutrition. The significant socio-economic covariates were in line with studies previously conducted in different countries [4–10, 44, 45, 49]. Particularly, our studies revealed that the risk of having CIAF decreased with an increase in the proportion of mothers’ education, which is in line with the results of previous works [4, 5, 10, 39, 50]. This might be due to the fact that educated mothers could feed their children better, as they have more knowledge, attitude, and practices (KAP) on nutrition-rich foods and the importance of a hygienic living environment [51]. The household wealth index is also found to be strongly associated with CIAF and children from the lowest (poorest) households are considerably disadvantaged concerning the CIAF than those from rich households. This is expected since poor households have no economic power to access and afford the required nutrition-rich foods and to access healthcare services, unlike their rich counterparts. In line with our findings, the role of household wealth status in undernutrition has also been well-documented in the extant literature [4, 16, 52–54, 3–5]. Even though the CIAF risk decreased from time to time, it is still high with increased trends in several zones in Ethiopia, which should be given priority when intervention and planning are made. Besides these, the decision-makers of those zones (showing increasing/no progress of CIAF) should pay more attention to the potential causes of CIAF, and the most important control mechanisms should be undertaken. In this study, potentially, we explored a vast number of risk factors for CIAF, but other influencing unobserved or unknown heterogeneous factors in space and time dimensions may be missed.

## Conclusions

The data used in this study are obtained from four waves of Ethiopian DHS. Ethiopia is located in East Africa and divided into 11 regions and 72 administrative zones. Our findings identified variations in the undernutrition of children under five among the 72 administrative zones in Ethiopia over the periods from 2000 to 2016. In this study, four space–time dynamic spatiotemporal models were used to model the relationships between the CIAF and covariates among the administrative zones in Ethiopia. This study provides meaningful information from a spatial analysis of the effects of the neighborhood contexts on the CIAF in Ethiopian 72 zones. Our empirical results revealed that the general nesting space–time dynamic model is more suitable for characterizing the dependent nature of undernutrition (CIAF) in the administrative zones over time. In summary, there exist geographical differences in CIAF in Ethiopian administrative zones, which are influenced by various neighborhood contexts. Higher breastfeeding rate, a lower percentage of comorbidity, a higher percentage of minimum and above dietary diversity, a higher percentage of literacy, and a higher percentage of BMI of women, were positively associated with higher values of CIAF. However, a higher percentage of unimproved water and a higher percentage of unimproved sanitation facilities, a low percentage of women's autonomy, a higher percentage of the employment status of women, a higher percentage of wealth index, and higher values of precipitation were positively associated with higher proportions of CIAF. There is a need to reassess the policies aimed at reducing the child malnutrition status in Ethiopia’s administrative zones.

## Data Availability

The dataset used for the current study is available at the DHS program repository and the shapefile of the map of Ethiopia was accessed as an open-source without restriction from open Africa 2016 https://dhsprogram.com/data/available-datasets.cfm.
